# Empyema1Read before the Section of Surgery, Buffalo Academy of Medicine, November 7, 1893.

**Published:** 1894-01

**Authors:** John Parmenter

**Affiliations:** Professor of Anatomy and Adjunct Professor of Clinical Surgery, Medical Department, University of Buffalo; Surgeon to Fitch and Children’s Hospitals; Assistant Surgeon to Buffalo General Hospital; Fellow of the American Surgical Association, etc.; 116 North Pearl Street


Buffalo Medical and Surgical Journal

VOL. XXXIV. JANUARY, 1894. NO. 6.

### Original Communications.

### EMPYEMA.[1]

BY JOHN PARMENTER, M. D.,

Professor of Anatomy and Adjunct Professor of Clinical Surgery, Medical Department, University of Buffalo ; Surgeon to Fitch and Children's Hospitals ; Assistant Surgeon to Buffalo General Hospital ; Fellow of the American Surgical Association, etc.

OUR ideas of empyema have undergone much change in the last few years, in that we now appreciate, as we did not formerly, how many and varied are the etiological factors which produce this disease. This change is due, in large part, to bacteriological investigation and its lessons, with the result that we now have a very clear and probably definite understanding of the pathology of empyema. Fluid within the pleural cavity may become purulent from either direct or indirect infection, and right here it will be timely to adopt a classification of purulent pleurisies based upon etiological considerations, and that of Courtois-Suffit is, it seems to me, the completest and the simplest of any yet offered. He divides purulent pleurisies into two distinct groups, viz., *pure* and *mixed* forms. In the *pure* forms only a single microbe is the active agent, and this may be an ordinary pus-producing microbe, or it may be a specific microbe which is accidentally pyogenetic in character. In the *mixed* forms two or more kinds of microbes have invaded the pleural cavity, either simultaneously or in succession.

*Pure Forms of Empyema.*—1. Empyema from the pneumococcus. This forms about twenty-five per cent. of purulent pleurisies (fifty per cent. in childhood). Inasmuch as the pneumococcus exists normally in various cavities, it can be the cause of an empy-

1. Read before the Section of Surgery, Buffalo Academy of Medicine, November 7, 1893.

ema without a preceding pneumonia. In fact, the pneumococcus may cause abscess in various places in the human body.

2. Empyema from the streptococcus. This microbe is the common agent in suppuration, and as it exists normally in the body cavities and all around us, it is no wonder that it can occasion an empyema. This it does do, however, not usually primarily, but rather in the course of infectious diseases not due to the streptococcus itself, such as scarlatina or grip. Here, as elsewhere, the streptococcus varies in activity and virulence. It seems to reach the pleura through the lymphatics after having been developed in parts more or less remote.

3. Empyema from the bacillus tuberculosis. Effusions due to the bacillus tuberculosis are usually serous in character,—at least in the beginning. The change to a purulent effusion, as seen in cases where repeated aspiration has been made, is explained by infection of the tubercular foci with pus microbes, and their introduction into a cavity already changed by disease, and in this way changes the type of inflammation and the character of the effusion. Very often the bacillus tuberculosis cannot be discovered, (Ehrlich notwithstanding, he claiming that it can be demonstrated in all cases,) but the pus is highly infective. Therefore, while the presence of the bacillus is conclusive of the tubercular nature of the effusion, its absence does not have any significance. Tubercular pus does not easily putrefy, and in this particular differs from that of the other forms.

4. Empyema due to the encapsulated bacillus of Friedländer, and to the typhoid bacillus of Eberth. These forms are very rare.

*Mixed Forms of Empyema.*— 1. Empyema due to the pneumococcus and the streptococcus together. In these cases we have a preceding pneumonia, followed by pulmonary suppuration due to the streptococcus.

2. Empyema due to the typhoid bacillus and the streptococcus. Here, again, the streptococcus causes secondary infection.

3. Empyema due to the bacillus tuberculosis and the streptococcus, or the staphylococcus, or both together. Such forms are seen where a cavity has ruptured into the pleural space, and the pleura has become directly infected. In these cases even the tubercle bacilli can only occasionally be found.

4. Empyema, putrid or gangrenous. In addition to the streptococcus and the staphylococcus, the microörganisms of putrefaction are active agents in producing this form of empyema. These latter organisms enter through the respiratory passages and the parenchyma of the lung, and feed on the products of coagulation necrosis. Usually, this occurs in a sub-pleural portion of the lung in or about one of the fissures between the lobes of the lung. These saprophytic bacilli may, however, be introduced during aspiration, the wound having become septic. In these cases the pus is extremely fetid, thin, and contains large shreds of fibrin. The symptoms are always severe.

*Differentiation of the Various Forms of Empyema.*—Can these various forms be differentiated ? Is it necessary or desirable to do so ? In answer to the first question, it must be confessed at the outset that reliance upon so-called clinical symptoms alone is apt to be misleading, and that bacteriological investigation can alone determine conclusively the nature of the disease. (The possible exception of tubercular empyema must here be noted.) However, certain symptoms are significant. For example, given a case occurring in youth and following a pneumonia, and in which the symptoms are moderate, with a more or less even range of temperature, the probabilities are in favor of such an empyema being due to the pneumococcus. Again, empyema due to the streptococcus and the mixed forms of the same, are consequent upon diseases such as scarlatina, grippe, typhoid fever, and the like. They form the great bulk of purulent pleurisies in adult life and advanced childhood. Finally, tubercular empyema is the typical latent purulent pleurisy. There may be no febrile reaction, and the chest be very full of fluid. In these cases we find the pleura, oftentimes, very much thickened, and the lung in an atalectatic condition, as seen after operation. Family history is, of course, significant. To resume, then, while we may find symptoms affording some clue to the causation of an empyema, we can only be certain when such symptoms are corroborated by the results of a bacteriological examination. To the second question it may be replied, that it is always well to cultivate the power to interpret clinical manifestations fully and accurately, and particularly for those who have neither the skill nor the facilities for using bacteriology as an adjuvant in diagnosis. Furthermore, our haste to afford relief is measured, in part, by the nature of the cases ; thus empyema from the streptococcus, or putrid, purulent pleurisy, demands speedy and energetic treatment in the former cases usually, in the latter, invariably.

*Diagnosis.*—It would seem that physical diagnosis had become sufficiently developed and diffused among practitioners to insure a fairly accurate estimate of the nature of the commoner forms of thoracic disease, and yet empyema, in its most typical and pronounced form, is constantly overlooked or misinterpreted. The following remarks of DaCosta are full of truth and wisdom :

Were the chest more often carefully explored, we should cease to hear of patients, whose pleural cavity is full of pus, being pronounced incurable consumptives because they are emaciating and have hectic fever and clubbed nails ; or being treated for disease of the heart, on account of displacement of that organ, and of dyspnea and edema ; or being dosed with mercury for an imaginary disorder of the liver ; or being subjected to long courses of quinia and arsenic to check a rebellious ague, which the chilly sensations and paroxysms of fever at times simulate.

I have seen the disease mistaken for every one of those DaCosta enumerates. I recall, particularly, one case in which the diagnosis was made of neuralgia of the liver, and that, too, by a practitioner of good average ability, who had evidently been misled by the fact that the pain was over the liver, and that a normal temperature existed, and yet I withdrew five quarts (by careful measurement) of pus from her right pleural cavity. Equally glaring mistakes have, doubtless, been noted by many who have seen cases as counsel. Such being the case, we should learn carefully the signs which indicate pleural effusion. The classification of signs as given by Powell seems to me good. It is :

1. Cardinal signs :

(*a*) Percussion dulness ; (*b*) displacement of the heart ; (*c*) annulled vocal fremitus ; (*d*) diminished and altered or absent breath sound.

2. Subordinate or supplementary signs :

(*a*) Increased semi-circumference of chest ; (*b*) intercostal bulging, elasticity or fluctuation ; (*c*) skodaic resonance ; (*d*) altered voice sound ; (*e*) displacement of abdominal viscera ; (*f*) signs in the other lung ; (*g*) cardiac displacement bruits.

3. Signs indicative of nature of fluid :

(*a*) Pectoriloquie aphonique (Bacelli) ; (*b*) temperature signs ; (*c*) other pyrexial or septic phenomena.

The presence of the cardinal signs is alone necessary for diagnosis. They are common to both serous and purulent effusions.

The subordinate signs are not essential for diagnosis, and any or all of them may be wanting.

A few words about some of the more important of the signs:

*Dulness.*—The dulness is absolute and toneless. It is flatness. Its limits are determined by light percussion, thus avoiding the resonance of contiguous parts. These limits, of course, vary with the amount of the effusion. When moderate, there is usually a triangular space of resonance in the upper part of the thoracic cavity, the apex of the triangle being at the sterno-clavicular joint, and the upper level of the effusion, instead of being a water level, is a slanting line, which, beginning at the sternal border, runs gradually upward and across the chest, to reach its highest level in the axillary line, where it begins to descend in its course across the posterior portion of the cavity, in a line similar to and slightly lower than anteriorly. When the effusion is extreme, flatness is, of course, universal. The lower limit of the effusion, where the same is even considerable, corresponds with the arch of the diaphragm, which is still preserved.

*Displacement of Heart toward the Sound Side.*—This sign must always be present, except in rare cases where the heart has become fixed by adhesions of the pericardium, consolidation of the opposite lung, etc. It occurs immediately, and keeps pace with the effusion. It is sometimes difficult to fix the exact location of the heart, but this can usually be done by percussion and ausculation.

*Absence of Vocal Fremitus.*—This is due to the presence of the fluid between the lung and the chest wall. This fluid is a bad conductor. To determine this sign, light palpation should be employed, using the finger tips only to better exclude vibrations from neighboring parts.

*Absence of Breath Sounds.*—While this sign may be, and usually is, a very significant one, it may be wanting, or, to put it in another way, the breath sounds may be present and a large effusion as well. When present, they may closely resemble those of pneumonia. Voice sounds, as well, may be conducted over the whole side, and whispering exaggerated even to pectoriloquy, according to Powell.

Subordinate or supplementary signs:

*Increased Semi-circumference of the Chest.*—Although the chest generally looks larger on the affected side, there may be little or no difference to actual measurement. The relative enlargement, when present, is increased during deep expiration. It must be remembered, however, that the total circumference is always increased in effusion. When the affected side is markedly larger than the sound, it denotes a large effusion. The shape is changed, the cystometer showing it to be more rounded.

*Intercostal Fulness or Fluctuation.*—This is an inconstant sign, more often found in children than in adults. It is more common in purulent than in serous effusions.

*Egophony* occurs very often in pleural effusions, and is chiefly of use in differentiating this condition from consolidation, which so often have a resemblance to each other.

*Skodaic Resonance.*—This sign is of value as an index of the pressure within the thorax and of the advisability of operative interference. When the pleural cavity is completely filled with fluid, skodaic resonance is, of course, abolished.

*Displacement of Abdominal Viscera.*—This sign occurs late in the disease, and is more often found when the effusion is purulent than serous. It is naturally caused by the descent of the diaphragm from the pressure of the fluid. The fact must, however, not be lost sight of that a very considerable effusion may coëxist with a normal situation of the diaphragm, *i. e.*, the stomach note may be obtained at the sixth rib in the nipple line, and yet a large effusion be present. What is true of the stomach note is equally true of liver dulness, and hence of importance, as otherwise injury to the viscera could easily occur during operation. The practical significance of this sign, therefore, varies. When present, it has a distinct diagnostic value. Its absence means nothing.

*Signs in the Other Lung.*—These consist of crepitant rales, cough, with more or less bloody sputum. They denote pronounced intra-pleural pressure, and are, therefore, to be found in cases where the effusion is very large. They, furthermore, indicate the necessity for withdrawal of the effusion.

*Cardiac Displacement Bruits.*—This sign is present when the heart is greatly displaced. It is usually a systolic murmur, developed over the base, and is supposed to be due to "straightening or slight tension of the great vessels from pressure of the fluid." (Powell.)

The signs which have just been enumerated are those of serous pleurisy as well as of empyema, but they belong no more to the one than other disease. Can we go further and find signs which are indicative of the nature of the fluid, or, to narrow the inquiry, indicative of pus in the thorax? Before enumerating the symptoms and signs which are of some value in determining the nature of the effusion, let me say that, in the earlier stages of empyema there are *none* that are pathognomonic. In other words, serous pleurisies may simulate, in every particular, an empyema of recent duration. Therefore, when a patient presents symptoms which are more or less adynamic, has rigors and chills which constantly recur and are followed by fever, has a very frequent pulse (120–140) with furred or dry tongue, anxious look and more or less rapid emaciation, and hectic sweats, which occur whenever the patient falls asleep, and has the physical signs which have been mentioned as characteristic of effusion, we may strongly suspect an empyema; but still, I repeat, each and every one of the symptoms and signs, in their full degree of development, may be met with in a serous pleurisy. In long-standing empyemata, however, there are a few signs which have an almost positive value.

*Edema of the chest wall* is one of these. Powell believes it to be an absolute sign of pus.

*An erysipelatous blush* over part of the chest and *pointing* are also sure signs of a purulent effusion, according to the same authority.

*Pectoriloquie Aphonique, or Bacelli's Sign.*—While this sign is of value, it is by no means pathognomonic. It is obtained as follows: the unaided ear is applied over the region beneath the scapula, and the patient made to whisper some rough word. This is conducted distinctly to the ear in serous effusions, but not when they are purulent, the difference being due to the fact that vibrations are better conducted through a homogeneous medium, like serum, than through a fluid, like pus. This sign occurs, however, too often in purulent effusions to regard it in any way an important one in differential diagnosis.

From what has been said, it is clear that we have no pathognomonic symptoms and signs of empyema at the time when we need them most, viz., early in the disease. There is but one way in which to ascertain whether an effusion be serous or purulent, and that is to use the exploring syringe. With this we can make a certain diagnosis; without it we can not. While I believe that the exploring needle should always be used, I do not think it should precede a careful consideration of all the symptoms and signs which have been mentioned. To be sure, when done in an aseptic way, the puncture is without danger; still, it is dreaded to some extent by the patient, but what is, in my opinion, more important, its early use leads us to ignore the clinical history, and causes us to disregard such evidences of the disease as demand careful study and interpretation. In short, each case should be so well studied that the diagnostician has come to a conclusion as to the probable nature of the effusion, and then uses his exploring needle to confirm his suspicions.

*Indications for Operation.*—These are two. First, to save the patient from impending death; and, second, to cure the disease.

*Contra-indications for Operation.*—Cases in which there is coëxisting and advanced phthisis, and certain hopeless cases of putrid or gangrenous empyema, are, so far as I can recall, the only ones in which a radical operation is contra-indicated. Not infrequently we see cases in which death seems imminent either from suffocation or cerebral anemia. It is easy to understand how suffocation can occur from the pressure of the fluid upon the lung of the opposite side, seriously limiting its expansion and that of the whole thorax as well, and causing congestion of the sound lung, which becomes less and less able to perform its function, until finally asphyxia occurs. It is not so clear how cerebral anemia is caused. Trousseau ascribed the cause to the dragging of the heart from its position, which, in turn, caused a sharp twist in the aorta. As a result of this condition, some sudden movement on the part of the patient could easily cause either an abrupt and complete interruption of the circulation, or rapid clotting and thrombus formation in the vascular system, and thus suddenly deprive the brain of blood. Bartels, on the other hand, would give a different explanation. He does not believe that the elastic aorta, imbedded as it is in connective tissue, can thus easily suffer torsion. According to this author, sudden death from cerebral anemia occurs only in cases where the effusion is upon the left side. When the heart is pressed out of its place, and to the right side, the aorta, as it comes through its opening in the diaphragm, suffers a sharp "kink," so to speak, this condition being aided by the fact that the aorta is quite firmly fixed in this situation. The kinking may amount to very nearly a right angle. If the inferior vena cava has a similar "kink," and the patient makes some sudden movement, or has a severe coughing fit with coincident spasmodic contraction of the diaphragm, then the heart, already somewhat empty of blood from the rapidly increasing exudate (especially in serous pleurisy), becomes suddenly so deficient in blood that the blood pressure is not sufficient to furnish the brain with arterial blood, and, according to the duration of this condition, the patient either faints or dies. Whichever explanation we may accept, one thing is certain : there is danger in delay, and especially in left-sided effusions with symptoms of congestion, as Trousseau has wisely observed. In fact, the indications for operation, where conditions like those just named exist, are as clear and imperative as the ligature of a bleeding vessel. Before, however, considering the operative treatment of empyema, a word or two about the medical or expectant plan of treatment. Experience has taught us, over and over again, that drugs will not remove pus from the pleural cavity. The best, therefore, that we can hope for is that Nature may come to the rescue, and the pus be discharged either through some point in the thoracic wall or through a bronchus. This happens only in most exceptional instances, and cannot, therefore, be depended upon to free the patient from so dangerous a malady as empyema becomes when allowed to go on indefinitely. Furthermore, even when natural discharge of pus does occur, caseous masses remain, which are highly infective, and which can and do cause general tuberculosis.

When shall we operate ? We have already seen that delay brings certain dangers, such as suffocation and cerebral anemia. Then, furthermore, we must remember that pus is a dangerous foreign body in itself, and that the longer it remains in the pleural cavity the more thickened the pleura becomes, and the more unable is the lung to expand. In addition to these unfavorable conditions can be added that of changes in the thorax wall (bones and cartilages), which may render final and complete closure of the cavity impossible.

*Methods of Operation.*—Few surgeons of experience, I imagine, now rely upon aspiration for the cure of empyema. Now and then a case occurs where one or two aspirations are sufficient to remove pus, which does not reaccumulate, and speedy cure ensues. Such results are extremely rare. Aspiration only succeeds, as a rule, where the empyema occurs in childhood and is due to the pneumococcus. Withdrawal of the pus, wholly or in part, is usually followed by increase of temperature, due, probably, to absorption of the pus, which absorption is stimulated by the relief of pressure, as is so commonly seen in serous pleurisy. Very soon the pleural cavity becomes again filled with pus, and the condition of the patient either unimproved or worse than before. As this is the usual history of cases in which aspiration is employed, it naturally does not commend itself to us as a safe and thorough method of treatment.

*Buelau's Method.*—This is a modification of the aspiration method of treatment. The pleural cavity is punctured with a large trocar, in the canula of which is introduced a long rubber tube. This runs down into a vessel of sublimate solution situated upon the floor. The end of the tube is weighted with a piece of lead, to keep it in the antiseptic solution. The object is to cause a negative pressure in the pleural cavity, by which the aspiration of the pus outward is favored. This treatment has received the favorable recommendation of Curschmann, Immerman, and Schede, the latter going so far as to say that the method would be ideal were it not for the ease with which the tube becomes displaced, especially in children and restless patients. On the other hand, it is claimed, and most judiciously, that this method is not applicable to putrid pleurisies or those due to the streptococcus, in which irrigation is so very commonly required. One distinct advantage it may have, as Duplay and Reclus have pointed out, and that is in cases of double empyema, where, from the fact that no air enters the pleural cavity, it would be a safer procedure than open incision, which, by admitting air with its attendant collapse of the lungs, would cause fatal asphyxia. It must, however, be confessed that Bülau's method, like simple aspiration, cannot completely empty the pleural cavity. The often huge caseous masses remain, and, although they may be reduced in size, something remains, and that something is highly infectious. It has been claimed that, even when aspiration cures the empyema, the patient invariably succumbs to tuberculosis within three years. This may or may not be true.

*Incision and Drainage.*—This, the most commonly employed procedure for the relief of empyema, has many things to commend it. It is easy to do, it is not dangerous, and, ordinarily, it accomplishes its purpose.

*Where Shall We Incise?*—The rule to open an abscess at its lowest point, thus favoring drainage, applies with equal force in the case of empyemata. The real difficulty is in determining what is the lowest point. This must, of course, vary with the position of the patient, so that, in choosing the site of our incision, we must settle upon what position the patient will take most constantly during the time when drainage is most important, viz., the first few days (seven to ten) after operation. This position will, most probably, be the dorsal, or inclining to the affected side. In such position, the lowest point in the thoracic cavity will correspond pretty accurately to the mid-axillary line or just in front of it, at about the sixth intercostal space. In addition to the advantage of drainage, the thoracic walls are here quite thin, so that the operation can be more easily performed than posteriorly, where the muscles are deep and the intercostal spaces narrow, making insertion of the tube and resection of a rib (where necessary) often quite difficult. Again, when the lung begins to expand, it touches the posterior walls first, and in this way an opening behind the axillary line may easily become occluded by the expanding lung and prevent free drainage. The diaphragm may do this same when the opening is made too low. The occurrence of the above undesirable conditions will induce us, then, to regard the fifth or sixth intercostal space, in or slightly in front of the mid-axillary line, as the best in which to incise and drain an empyema.

*The Operation.*—The skin over the region having been thoroughly disinfected and the patient brought well to the edge of the table, in order that he may lie as nearly on his back as possible and still give room for the surgeon, the arm of the affected side, is raised to a right angle, and the incision (which should be from one and one-half to three inches long) made at the level of the upper border of the lower rib of the intercostal space selected. The position of the arm is important, inasmuch as lifting the arm causes the skin to slide upward upon the thoracic wall; and if the amount of this displacement be not noted, the incision in the skin and that in the intercostal muscle will not be in the same plane when the arm is lowered, and thus the opening will be a valvular instead of a direct one. When the pleura has been reached, a grooved director is thrust through the pleura and the opening further widened with a pair of forceps. The pus is then allowed to escape slowly and the expulsion of caseous masses facilitated with the finger introduced into the cavity. Should examination show that a counter opening is advisable, this can easily be made by introducing some stiff instrument, such as long forceps or a sound, and cutting down upon the same at the point over the end of the instrument. A drainage-tube is then inserted in as many openings as are made, *i. e.*, one for each opening.

When it is found that the intercostal spaces are too narrow to permit the introduction of a good-sized drainage-tube, or the finger, for examination purposes, it is well to resect a portion of one or two ribs. The rib is bared of its periosteum, and the intercostal artery avoided, and then, being steadied with forceps, it can be divided with a fine saw, or partially divided, and the section finished with cutting forceps. The ribs should not be divided with forceps alone, as they are easily splintered in this way. After an inch, or more, of rib has been resected, the periosteum should be removed, as its retention may easily lead to an irregular callous formation.

*Form of Drainage-tube.*—The tube should be of good size, flexible, and just long enough to penetrate completely the thoracic wall. A longer tube very soon begins to fret against the expanding lung and to cause distress, if not positive injury. To prevent an accident which has happened very often, viz., dropping of the tube into the pleural cavity, some expedient must be resorted to, to anchor the tube in place. Personally, I am quite partial to the Baxter tube. It is made as follows: Take a piece of sheet India-rubber, one and one-half to two inches square, and punch in the center of it a hole as large as the drainage-tube. Pass the tube through and split it into four pieces long enough to reach to the four corners of the shield, and fastened or sewed to the same with fine silver wire. The tube, on the other side of the shield, should have no holes cut in its side, as granulations can easily enter them and distress the patient or occlude the tube.

### THE AFTER-TREATMENT.

*Shall We Irrigate the Cavity?*—In the vast majority of empyemata, irrigation is unnecessary. It may be injurious. Many cases are on record in which the antiseptic solution employed has caused severe toxic symptoms and even death. Again, the injection fluid, striking against the pericardium, or from some unknown cause, has caused sudden arrest of the heart's action with fatal result. This is the more apt to happen when the fluid has been too cool when used. Irrigation is, then, to be employed only in exceptional cases, such as putrid empyemata with severe symptoms. When employed, irrigation should be done with a fountain syringe, raised about a foot above the level of the cavity, or, at least, the fluid made to enter the cavity slowly and steadily. The amount allowed to enter at one time is, of course, determined by the size of the cavity. The sensations of the patient are a reliable guide, and the first sign of discomfort made the signal for ceasing. The solutions used to wash out the pleural cavity have been many and various. In general, it may be said that the stronger solutions are not to be used. Carbolic acid, in any strength, ought not to be employed. Sublimate, 1–4000, and followed by boiled water, makes an excellent injection. My own preference, however, is for iodine, which should be added to the water until it has a light sherry-red tint. The amount and number of the irrigations must vary with each individual case. Ordinarily, to flush out the cavity once each day suffices, but now and then a case will occur when it should be done three, or even more times, in twenty-four hours.

The dressing should be the ordinary one of iodoform, bichloride gauze and cotton, held firmly in place with a well-fitting bandage. It should be changed as soon as the discharge appears through it and as often. This usually means twice during the first twenty-four hours and then at gradually increasing intervals. The regulation of the diet and the exhibition of reconstructive tonics will follow in due course. Just about this time, it is not infrequent to see patients who have been doing well, in every way, develop an acute indigestion, quite violent in its symptoms. Such manifestations must be met in an appropriate way, but particularly must the cause be removed, viz., feeding in excess of the patient's powers of assimilation. A patient with a pleural cavity filled with pus, and breathing forty to sixty times per minute, needs a generous supply of food to compensate for the waste due to the rapid oxidation going on within his body. Lessen the oxidation by restoring the respiratory process more nearly to the normal, so should the amount of food become proportionately decreased. So anxious for his recovery, however, are relatives, and too often the physician himself, that food is constantly urged upon the patient to his, at least, temporary detriment.

When may the tube be removed? This is frequently one of the most puzzling questions that will arise in the treatment of empyema. So long as a distinct cavity remains, the tube should be retained. Usually, when the discharge has become serous and small in quantity, it means that the cavity has become obliterated. This change of the discharge from purulent to serous occurs at varying times, from two weeks to months, and sometimes years, depending upon the age, resistance of the chest wall, expansibility of the lung, etc. Not long ago I removed a quart of pus from the chest of a boy of five years, and on the sixteenth day after operation the tube was taken out and the wound allowed to close. It must be remembered that the wound itself gives rise to some pus, which, mixing with the serous discharge, could easily deceive the over-anxious. Before removing the tube, it is good practice to carefully examine the cavity with a probe, and thus ascertain definitely the form and extent of the same. Too often trouble arises from removing a tube at an improper time. This improper time may be too late as well as too early, for, while we all appreciate the danger of locking up purulent secretions by taking away their means of exit, we do not so often remember that, by leaving a tube in too long, we are apt to help the formation of long sinuses, which may be very troublesome to heal.

*Accidents During Operation.—Hemorrhage* may occur. When it does, it is usually due to a wound of the intercostal artery, at the site of operation, and always due to the lack of skill of the operator. It usually is caused by a partial severing of the artery. This can frequently be remedied by cutting through the artery down upon the rib with a tenotonic, in this way making a clean division, when the edges of the vessel curl up and the hemorrhage ceases. This failing, a hemostatic can be clamped upon the vessel and left in position for twenty-four hours, or a small portion of the rib exsected and the artery secured with a needle and catgut. Usually, the accident is more annoying than serious, but occasionally the bleeding may be severe enough to cause alarming symptoms.

*Wounding of the Diaphragm.*—It has already been mentioned that a large effusion may be present without appreciable descent of the diaphragm, and it was hinted that, unless this fact were remembered, an undesirable accident might occur. Such an accident occurred to me some three months ago. In operating, without assistance, upon a child of sixteen months, with left-sided empyema, and in which the pressure signs were so urgent that haste was demanded, I used a bistoury and opened the chest wall with one cut, in the sixth intercostal space (axillary line). Over a pint of pus escaped, and was followed by a portion of the great omentum. The child made a good recovery, and without any unpleasant symptoms, but died six weeks later of pneumonia affecting the sound lung. While in this case nothing untoward resulted, one does not enjoy opening the peritoneal cavity in the presence of pus, or incising the liver under similar conditions.

Wounding the diaphragm is, then, a possibility, and due care should be taken to ascertain its limits before operation.

*Nervous Phenomena.*—Epileptiform convulsions, various kinds of paralyses, and sudden death have followed irrigation of the pleural cavity, so that, when this is employed, the above-mentioned possibilities must be borne in mind and the injection most carefully given.

The limits of this paper demand that mere mention only be made of the more radical methods which may and have been used in those obstinate cases in which, from one cause or another, the pus cavity cannot be obliterated by simple incision and drainage, with or without resection of the rib.

*Estlander's Operation.*—This consists, essentially, in the resection of several ribs, a greater or less length of rib being removed, according to the size of the cavity. I pass by the details of this operation, and say of it simply that it often fails in its purpose, unless accompanied with curetting of the pleura or more or less extensive resection of the same. In moderately severe cases it is of decided value, but, given a large cavity, with rigid pleura and a very contracted lung, not much can be expected from this procedure.

*Quenu's Operation.*—Quenu aims to get a movable chest wall by resecting the fifth and sixth ribs anteriorly and posteriorly, the intervening part acting like a mobile shutter, as it were. It can be easily understood how great a hindrance to the success of this method a rigid and thickened pleura must be, and, therefore, it has serious limitations in those very cases where it is most needed.

*Schede's Operation.*—More radical than either of the before-mentioned operations is that of Max Schede. This surgeon resects the entire thoracic wall, excepting only the superficial parts. The ribs, intercostal muscles, and the pleura are all removed, and the large flap put in opposition to the lung. The disadvantages of the operation are obvious. The large flap does not fit accurately the lung underneath, and often leaves part of the cavity exposed; cicatrization is often tardy and imperfect, and the thorax, as a whole, left in a weakened condition.

*Prognosis.*—The great majority of cases recover, and do so in a space of time which varies from three weeks to two months. The time has been materially shortened since the introduction of the antiseptic method of treatment and the discontinuance of irrigation. The worst results will be found in patients of more advanced age, in which the thoracic walls have become too rigid to yield; in tuberculous subjects, where an unduly thickened pleura prevents expansion of the lung; in long-neglected cases, and in the putrid or gangrenous varieties. In such cases, more heroic treatment, as has just been mentioned, will often be required than simple incision and drainage; but, even in these, one should not despair, as experience teaches us every day most convincingly how much Nature can accomplish if she receives adequate, timely, and otherwise judicious assistance.

116 North Pearl Street.

